# Phenotypic and Genotypic Correlates of Penicillin Susceptibility in Nontoxigenic *Corynebacterium diphtheriae*, British Columbia, Canada, 2015–2018

**DOI:** 10.3201/eid2601.191241

**Published:** 2020-01

**Authors:** Jason Zou, Samuel D. Chorlton, Marc G. Romney, Michael Payne, Tanya Lawson, Anna Wong, Sylvie Champagne, Gordon Ritchie, Christopher F. Lowe

**Affiliations:** St. Paul’s Hospital, Vancouver, British Columbia, Canada (J. Zou, M.G. Romney, M. Payne, T. Lawson, A. Wong, S. Champagne, G. Ritchie, C.F. Lowe);; University of British Columbia, Vancouver (S.D. Chorlton, M.G. Romney, M. Payne, S. Champagne, G. Ritchie, C.F. Lowe)

**Keywords:** Corynebacterium diphtheriae, diphtheria, whole-genome sequencing, penicillin, antimicrobial susceptibility, phenotypes, genotypes, antimicrobial resistance, bacteria, AMR, British Columbia, Canada

## Abstract

In 2015, the Clinical and Laboratory Standards Institute (CLSI) updated its breakpoints for penicillin susceptibility in *Corynebacterium* species from <1 mg/L to <0.12 mg/L. We assessed the effect of this change on *C. diphtheriae* susceptibility reported at an inner city, tertiary care center in Vancouver, British Columbia, Canada, during 2015–2018 and performed whole-genome sequencing to investigate phenotypic and genotypic resistance to penicillin. We identified 44/45 isolates that were intermediately susceptible to penicillin by the 2015 breakpoint, despite meeting previous CLSI criteria for susceptibility. Sequencing did not reveal β-lactam resistance genes. Multilocus sequence typing revealed a notable predominance of sequence type 76. Overall, we saw no evidence of penicillin nonsusceptibility at the phenotypic or genotypic level in *C. diphtheriae* isolates from our institution. The 2015 CLSI breakpoint change could cause misclassification of penicillin susceptibility in *C. diphtheriae* isolates, potentially leading to suboptimal antimicrobial treatment selection.

Since the introduction of the diphtheria toxoid vaccine, cases of diphtheria caused by toxigenic strains of *Corynebacterium diphtheriae* have decreased (*1*). More recently, however, *C. diphtheriae* appears to have reemerged, with outbreaks of diphtheria occurring globally and with increasing frequency. In the 1990s, states in the former Soviet Union experienced several epidemics (*2*). Since 2010, outbreaks have been described almost yearly and span the globe, including South America (*3*,*4*), Southeast Asia (*5*), South Africa (*6*,*7*), and Europe (*8*,*9*). Data from national surveillance programs, such as one in Latvia, have shown that diphtheria incidence can increase despite adequate vaccination programs (*10*). Furthermore, serologic studies performed in Europe show that waning or inadequate immunity to diphtheria is becoming more common, indicating populations increasingly are susceptible to diphtheria reemergence (*11*,*12*).

Those living in impoverished, urban settings, even in developed countries, appear to be especially susceptible to *C. diphtheriae* infection. Nontoxigenic strains have been shown to have epidemic potential, causing infections in persons afflicted by homelessness, alcohol abuse, and injection drug use (*9*,*13*–*15*). Nontoxigenic strains of *C. diphtheriae*, against which the toxoid vaccine does not provide immunity, are being reported with greater frequency as a source of severe disease, both in the form of cutaneous diphtheria and more invasive infections, such as bacteremia and endocarditis (*14*–*21*). In addition, nontoxigenic strains have the potential to become toxigenic through exposure to corynebacteriophages carrying the toxin gene, particularly through contact with toxin-producing strains carried by travelers returning from diphtheria-endemic countries (*22*). Use of available, effective, and well-tolerated antimicrobial drugs targeted to *C. diphtheriae* infection can counter the growing threat.

Currently, penicillin and erythromycin are considered first-line antimicrobial drugs for diphtheria treatment (*23*). Since 2010, a limited number of case reports from Canada, the United States, and the United Kingdom have described *C. diphtheriae* isolates resistant to penicillin and other conventional antimicrobial drugs (*24*–*26*). In 2015, the Clinical and Laboratory Standards Institute (CLSI) lowered the penicillin-susceptible breakpoint for *C. diphtheriae* from a MIC of <1 mg/L to <0.12 mg/L, citing expert opinions and discordance with breakpoints determined by the European Committee on Antimicrobial Susceptibility Testing as reasons for the change (*27*,*28*). With the change, many *C. diphtheriae* isolates previously considered penicillin-susceptible are now classified as penicillin-intermediate (i.e., intermediately susceptible to penicillin). Consequently, clinicians might opt for alternative antimicrobial drug regimens, such as clindamycin, vancomycin, or erythromycin, the alternative first-line agent. However, these drugs are limited by gastrointestinal side effects, increased risk for *C. difficile* infection, and unnecessary broad-spectrum antimicrobial exposure.

No published reports have demonstrated whether the reclassification of some *C. diphtheriae* isolates to penicillin-intermediate truly reflects an increasing prevalence of penicillin resistance at the phenotypic and genotypic levels. Limited evidence of penicillin-resistant *C. diphtheriae* infections have been reported in cases in which failure of initial penicillin therapy necessitated a change to broad-spectrum antimicrobial drugs before the patients’ clinical signs and symptoms improved (*24*,*26*). The absence of large-scale susceptibility testing leaves a scarcity of data. One available study reviewed susceptibility testing performed on ≈200 *C. diphtheriae* isolates collected from various provincial reference laboratories across Canada during 2006–2015 and found 100% of isolates tested were susceptible to penicillin, as defined by a MIC of <1 mg/L (*29*).

*C. diphtheriae* is a reemerging pathogen of public health concern and penicillin breakpoint changes could have implications for clinical treatment. We assessed the evolving trends in *C. diphtheriae* antimicrobial nonsusceptibility at the phenotypic and genotypic levels by performing susceptibility testing and whole-genome sequencing (WGS) analysis on isolates collected in Vancouver, British Columbia, Canada, during 2015–2018.

## Materials and Methods

### Collection of *C. diphtheriae* isolates

We isolated *C. diphtheriae* from blood, throat, and wound cultures collected from inpatients and outpatients at St. Paul’s Hospital, an inner city, tertiary care center in Vancouver, during March 2015–September 2018. We included all unique *C. diphtheriae* isolates. For patients with multiple *C. diphtheriae* isolates during the study period, we only included the first isolate. One patient in the study had 2 isolates recovered the same day from blood and wound cultures; we only obtained MICs from the blood isolate, but we performed WGS on the wound isolate, assuming the 2 isolates would represent the same strain. We confirmed *C. diphtheriae* isolates by using methods described previously (*14*). The National Microbiology Laboratory of the Public Health Agency of Canada confirmed all isolates were nontoxigenic by using a modified Elek test and PCR. We obtained ethics approval for this study from the University of British Columbia−Providence Health Care Research Ethics Board.

### Antimicrobial Susceptibility Testing and Interpretation

We performed antimicrobial susceptibility testing for penicillin, erythromycin, clindamycin, and vancomycin by using Etest (bioMérieux, https://www.biomerieux.com). We interpreted results by using breakpoints from the second edition of the CLSI M45 guidelines (*30*) published in 2010 and from the third edition (*27*) published in 2015.

### WGS, Multilocus Sequence Type, and Antimicrobial Resistance Marker Analysis

We stored *C. diphtheriae* isolates in trypticase soy broth with 13.8% glycerol at −70°C. For WGS, we thawed and subcultured the isolates onto 5% sheep blood agar plates. We incubated single isolates in Mueller-Hinton broth at 37°C for 48 h. After incubation, we resuspended the cultures in phosphate buffered saline with 1% sodium dodecyl sulfate and 0.25 mg/mL proteinase K and incubated them overnight at 55°C before heating to 95°C for 15 min and bead lysing on the TissueLyser LT (QIAGEN, https://www.qiagen.com) at a setting of 50 for 2 min. We performed DNA extraction on the MagNA Pure Compact (Roche Diagnostics, https://www.roche.com) and eluted in 50 µL elution buffer.

We used the KAPA HyperPlus Kit with KAPA dual-indexed adapters (Roche Sequencing, https://sequencing.roche.com) for WGS. We assessed DNA library quality by using Agilent High Sensitivity DNA Chips on the Bioanalyzer 2000 (Agilent Technologies, https://www.agilent.com). After normalization of the samples, we sequenced DNA on the MiSeq (Illumina, https://www.illumina.com) platform using MiSeq Reagent 2×300 V3 Kit (Illumina). We submitted processing codes to GitHub (https://github.com/schorlton/cdip_sequencing). We preprocessed reads to remove low-quality and contaminated sequences, and then performed de novo assembly. We identified multilocus sequence types (STs) through analysis of assembled scaffolds by using the Center for Genomic Epidemiology database version 2.0.0 and multilocus sequence type (MLST) tool 2.0.1 (https://www.genomicepidemiology.org). We identified antimicrobial nonsusceptibility by using the Resistance Gene Identifier in the CARD database version 3.0.2 (*31**,**32*; http://card.mcmaster.ca) to locate individual markers of nonsusceptibility.

## Results

### Isolate Characteristics

We identified 60 nontoxigenic *C. diphtheriae* isolates during the study period, 1 from a blood culture, 1 from a throat culture, and 58 from wound cultures. We identified 4 isolates in 2015, 12 in 2016, 26 in 2017, and 18 in 2018. We conducted WGS on 56/60 (93.3%) isolates and obtained MICs from susceptibility testing for 45/60 (75%) isolates.

### Antimicrobial Susceptibility Testing

We obtained MICs by susceptibility testing for penicillin, erythromycin, clindamycin, and vancomycin (Figure). The MIC required for 50% growth inhibition for penicillin was 0.25 mg/L and for 90% growth inhibition was 0.38 mg/L. Using the 2010 edition of the CLSI breakpoints, all isolates were penicillin-susceptible. Using the 2015 edition of CLSI breakpoints, all isolates, except 1 with a MIC of 0.125 mg/L, were nonsusceptible (Figure, panel A). The distribution of MICs for penicillin did not change greatly over the study period. One isolate was resistant to erythromycin and clindamycin with a MIC of >256 mg/L for both agents (Figure, panel B). All isolates tested were susceptible to vancomycin (Figure, panel B). Interpretations for erythromycin, clindamycin, and vancomycin testing were unchanged between the 2010 and 2015 CLSI M45 breakpoints.

**Figure:**
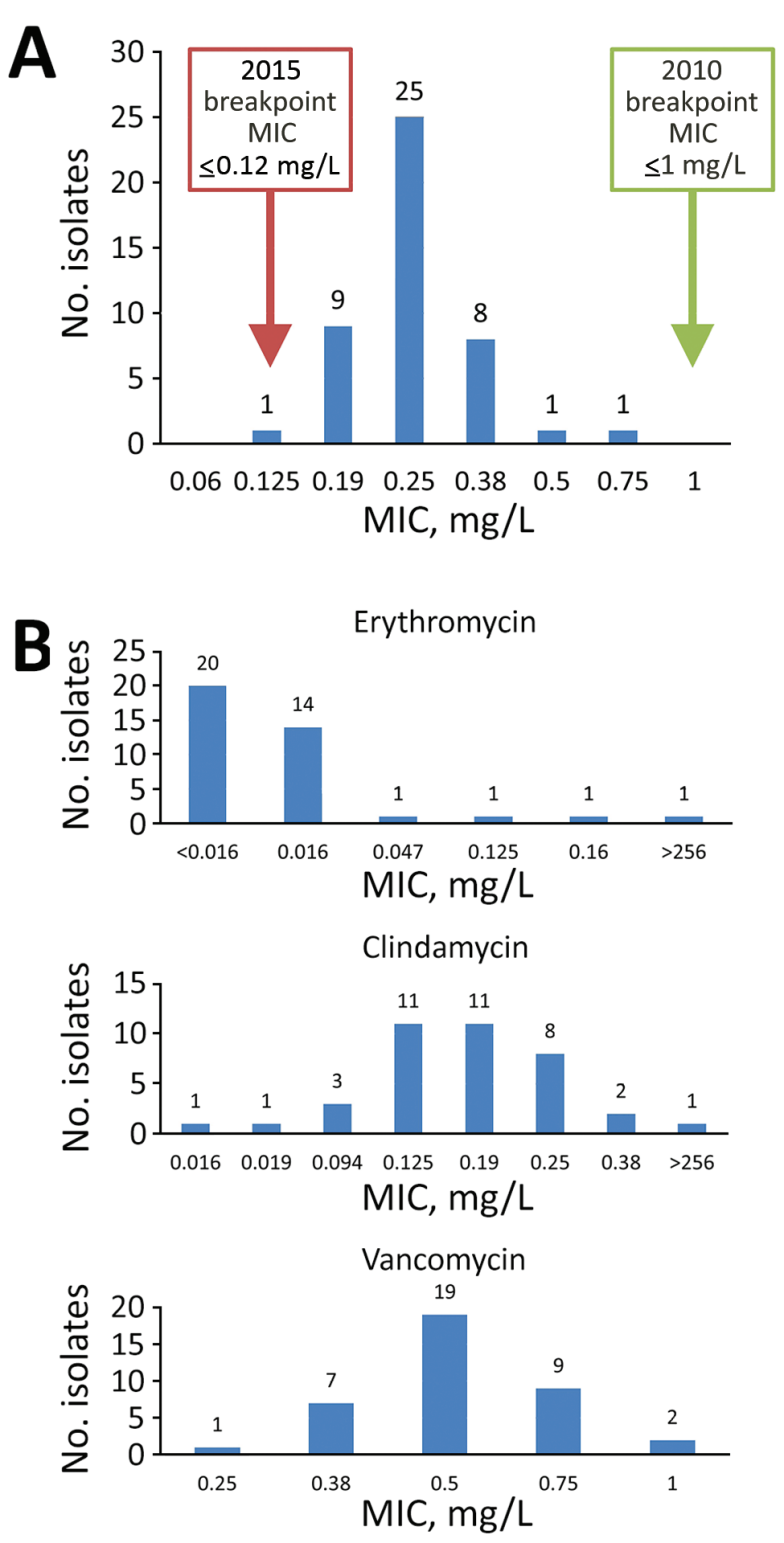
Distribution of MICs from antimicrobial susceptibility testing on *Corynebacterium diphtheriae* isolates collected at St. Paul’s Hospital, Vancouver, British Columbia, Canada during March 2015–September 2018. A) MICs from penicillin susceptibility testing. Green box indicates penicillin-susceptible breakpoints from the 2010 Clinical Laboratory Standards Institute (CLSI) M45 guidelines (*30*); red box indicates penicillin-susceptible breakpoints from the 2015 CLSI M45 guidelines (*27*). B) MICs for erythromycin, clindamycin, and vancomycin susceptibility testing.

### WGS Analysis, MLST, and Genotypic Correlates of Resistance

MLST typing revealed ST76 was the predominant strain in our study, in 52/56 isolates. We also noted 1 each of ST5, ST32, ST319, and 1 novel ST most similar to ST441/442/444.

We sequenced 56 isolates to investigate resistance markers, yielding a median of 0.4 million (interquartile range [IQR] 0.3–1.05 million) paired-end reads per sample and an estimated median coverage of 100× (IQR 75–263×) per isolate. Of the 56 samples sequenced, 27 had a mean read quality >30 before trimming; all samples had a mean read quality >24. We successfully assembled genomes for 45 study isolates, recovering a median of 59 contigs (IQR 55–62 contigs) and 2.388 Mbp (IQR 2.387–2.389 Mbp). We recovered 50% of each assembly length with contigs of 158.8 kbp (IQR 158.8–180.3 kbp) or longer. An additional 11 isolates had incomplete or fragmented assemblies, a median of 530 contigs (IQR 190–1,302 contigs), a length of 2.38 Mbp (IQR 2.086–2.398 Mbp), and an N50 (the length of the smallest contig among the set of the largest contigs that together cover >50% of the assembly) of 7.1 kbp (IQR 1.8–27.7 kbp).

In the fully assembled genomes, 39/45 isolates had the *sul1* gene, conferring sulfonamide resistance, 1 isolate had *tetO*, conferring tetracycline resistance, and 1 isolate had a plasmid harboring *ermX*, conferring macrolide and lincosamide resistance. The isolate carrying *ermX* exhibited phenotypic resistance to erythromycin and clindamycin during susceptibility testing with MIC of >256 mg/L (Figure, panel B). We detected *sul1* in 7/11 incomplete assemblies. None of the isolates we tested contained single-nucleotide variants in the CARD database, nor any markers of β-lactam resistance.

## Discussion

Reports of *C. diphtheriae* outbreaks are becoming increasingly common. In particular, nontoxigenic strains pose a major threat to public health because they are not targeted by the current diphtheria toxoid vaccine and can cause invasive infections. Most (44/45) *C. diphtheriae* isolates collected at our institution during 2015–2018 were reported as penicillin-intermediate in accordance with the updated 2015 CLSI M45 breakpoints. However, this reclassification of susceptibility does not appear to be supported by evidence of resistance to penicillin at the phenotypic or genotypic level, at least within isolates identified from our institution’s inner-city catchment area. In addition, the distribution of MICs for penicillin does not appear to have changed substantially over the 4-year study period.

Antimicrobial susceptibility testing revealed 2.2% of isolates in our study were erythromycin-resistant, but none were penicillin-resistant. These results are similar to those from a 2015 study in Canada by Bernard et al. (*29*) in which 32/195 (16.4%) isolates were erythromycin-resistant (MIC of >2 mg/L) by broth microdilution susceptibility testing, but none were penicillin-intermediate or penicillin-resistant (MIC of >1 mg/L).

Misclassification of penicillin susceptibility could have clinical implications. A preference for erythromycin over penicillin as a first-line therapy for *C. diphtheriae* infection could increase rates of inappropriate treatment because of greater rates of erythromycin resistance observed to date. Another disadvantage of using erythromycin for treating patients with cutaneous diphtheria is that wound cultures positive for *C. diphtheriae* often are concurrently positive for group A *Streptococcus*, for which penicillin is the optimal antimicrobial agent (*13*).

Maintaining effective antimicrobial options is essential to curtailing future outbreaks. The change in the breakpoint for penicillin susceptibility published in the CLSI M45 third edition in 2015 (*27*) could affect treatment decisions by clinicians. Clinical outcomes are unclear for patients with *C. diphtheriae* infection with MICs in the 0.12–1 mg/L range. A 2011 case report in Canada described a multidrug resistant *C. diphtheriae* isolate harboring the *ermX* gene and exhibiting resistance to erythromycin, clindamycin, and sulfonamide (*25*). The isolate had an MIC of penicillin of 0.25 mg/L and the patient ultimately was treated successfully with cephalexin (*25*).

Cases of penicillin treatment failure have been described in other reports. A case of *C. diphtheriae* endocarditis (MIC of >16 mg/L) was reported in a child who was refractory to initial therapy with penicillin G and whose condition did not improve until antimicrobial treatment was changed to meropenem and vancomycin (*24*). FitzGerald et al. (*26*) reported another case of penicillin G treatment failure in a child in the United Kingdom with cutaneous diphtheria. The patient’s isolate was later found to be nonsusceptible to penicillin, but the MIC was not reported. The patient recovered shortly after a macrolide was administered, but the treatment caused intense gastrointestinal side effects (*26*).

Overall, our study and other reviews of *C. diphtheriae* susceptibility performed in Canada and globally suggest that elevated MICs of penicillin are rare (*29*,*33*). Despite clinical observation of isolated cases of penicillin resistance, one could argue that prematurely opting for erythromycin as first-line therapy for *C. diphtheriae* infection poses a greater risk than penicillin, given the aforementioned concerns of erythromycin resistance and potential for increased adverse effects.

Of note, WGS did not identify genetic markers of β-lactam resistance in the study isolates. However, WGS did detect *ermX* in the isolate phenotypically resistant to erythromycin; *ermX* is a gene encoding a 23S rRNA adenine N-6-methyltransferase known to confer resistance to erythromycin and clindamycin in *C. diphtheriae* (*25*,*34*,*35*). Although the specific goal of sequencing was to detect β-lactam resistance mechanisms, WGS afforded the ability to identify other potential markers of antimicrobial resistance, namely *sul1* and *tet*. The *sul1* gene is known to encode dihydropteroate synthase, which can confer resistance to sulfonamides and the *tet* gene encodes a ribosomal protection protein that can mediate tetracycline resistance (*36*). A case of both *sul1* and *tet* genes in a nondiphtheria corynebacterial infection with corresponding elevated MICs of 32 mg/L was reported but has yet to be associated with *C. diphtheriae* (*37*).

Our laboratory does not routinely perform antimicrobial susceptibility testing for sulfonamides and tetracyclines because neither is recommended routinely for *C. diphtheriae* infections. The clinical relevance of detecting *sul1* and *tet* genes is unclear and requires further study. Additional susceptibility testing is needed to establish whether the study isolates carrying *sul1* and *tetO* exhibited corresponding phenotypic resistance to sulfonamides and tetracyclines.

ST76 was predominant in our study, exhibited in 93% of isolates. Further analysis confirmed that these isolates represented a single clonal strain (*38*), representing further clonal expansion compared with a previous study of *C. diphtheriae* isolates identified in Vancouver during 1998–2007. ST76 also was the dominant sequence type in that review but encompassed only 69% of isolates (*13*). ST76 has been reported elsewhere. An instance in an online MLST database, PubMLST.org (https://pubmlst.org/cdiphtheriae), lists 9 ST76 isolates submitted from St. Petersburg, Russia, during 2005–2010. All were nontoxigenic by Elek test. In another report, 5 isolates from Belarus collected during 2004–2014 also were typed as ST76 and found to be nontoxigenic (*39*). No epidemiologic links are apparent between our isolates and those described from Russia or Belarus.

We also noted ST5 and ST32 in our review from downtown Vancouver during 1998–2007 (*13*), although less frequently observed in our study. ST5 previously was recovered in Russia, the United States, and France, and ST32 is known to circulate in Europe and Australia (*13*,*33*,*39*,*40*). The novel sequence type related to ST441/442/444 identified in this study has not been described in other studies to date.

Among ST76 isolates, we noted heterogeneity in antimicrobial resistance marker carriage, specifically *sul1* and *ermX*, and 5/52 (9.6%) ST76 isolates had no identifiable markers. Patterns of resistance marker carriage also differed between isolates of other MLSTs, with no markers found in ST5 or ST32 isolates, *sul1* in the ST319 isolate, and *tetO* in the ST441/442/444 isolate. In addition to the 7 target regions used for routine MLST in our study, further assessment of intrastrain variability and the extent of clonality is warranted through analysis of additional genomic loci such as those performed in other studies of *C. diphtheriae* epidemiology (*9*).

Our study has some limitations. Our catchment area was small and limited to the persons from downtown Vancouver treated at St. Paul’s Hospital, and MLST indicated highly clonal *C. diphtheriae* isolates. The generalizability of our results is limited and further study is needed to understand how our findings apply to other patient populations with different *C. diphtheriae* epidemiology (*13*). Nonetheless, several recent studies in multiple regions appear to support the notion that penicillin resistance remains scarce in circulating strains of *C. diphtheriae*. Other reviews of penicillin susceptibility in *C. diphtheriae*, 1 in Canada on 195 isolates and 1 in Algeria on 157 isolates, reported none that would be considered penicillin nonsusceptible by the 2010 CLSI breakpoints of MIC >1 mg/L (*29*,*33*). 

Another limitation of our study is the lack of clinical outcomes to supplement our phenotypic and genotypic susceptibility data, precluding assessment of treatment success and failure rates on the basis of molecular and phenotypic characterization of isolates. Examination of clinical outcomes for patients treated for penicillin-intermediate diphtheria with MICs of 0.12–1 mg/L would be useful, as would an assessment of changes in prescribing practices for antimicrobial drugs related to increasing rates of isolates classified as penicillin-intermediate. Future studies are needed to explore clinical impacts of reclassification of isolates as penicillin-intermediate. 

Last, any study using next-generation sequencing techniques to identify known resistance markers from databases, such as our study, has certain biases and limitations inherent to the sequencing and marker identification process. Current sequencing studies cannot account for markers not yet identified in databases, nor can such studies identify mixed or unspecified genetic effects. In addition, regions with poor assembly quality or with sequence or GC-content-dependent assembly gaps can preclude database matching.

In conclusion, we report the absence of penicillin nonsusceptibility in *C. diphtheriae* isolates collected during 2015–2018 and assessed phenotypically by susceptibility testing and genotypically by WGS. These results indicate that the 2015 CLSI M45 guidelines lowering the penicillin-susceptible breakpoint from an MIC of <1 mg/L to an MIC of <0.12 mg/L for *C. diphtheriae* might misclassify penicillin susceptibility in isolates. Such misclassification could lead to shifts in prescribing practices toward less effective, less well-tolerated, and broader-spectrum antimicrobial drugs than penicillin. Further study is warranted to assess penicillin susceptibility in other contexts in which local strains and resistance patterns differ.
